# Quantitative imaging of chromatin inheritance using a dual-color histone in *Drosophila* germinal stem cells

**DOI:** 10.1016/j.xpro.2022.101811

**Published:** 2022-11-02

**Authors:** Rajesh Ranjan, Xin Chen

**Affiliations:** 1 Howard Hughes Medical Institute, Department of Biology, The Johns Hopkins University, 3400 North Charles Street, Baltimore, MD 21218-2685, USA; 2 Department of Biology, The Johns Hopkins University, 3400 North Charles Street, Baltimore, MD 21218-2685, USA

**Keywords:** Cell biology, Developmental biology, Microscopy, Model organisms, Molecular biology, Molecular/Chemical probes, Stem cells

## Abstract

We detail a stepwise protocol for the investigation and quantification of chromatin features during asymmetric cell division (ACD) of *Drosophila* germline stem cells (GSCs). We describe the use of a dual-color histone to study the inheritance of new and old histones. We detail steps for imaging and analysis of sister chromatid condensation dynamics and nucleosome density changes. In addition, this protocol could be applied to identify stem cells, which can be challenging to identify in intact tissues.

For complete details on the use and execution of this protocol, please refer to Tran et al. (2012), Ranjan et al. (2019), and Ranjan et al. (2022).

## Highlights

• Generation of dual-color histone to study inheritance pattern of old and new histones
• Dynamics of chromatids condensation and nucleosome density during stem cell division
• Detailed for *Drosophila*, but can be applied to other organisms to identify stem cell

## Abstract (editor highlights)

Publisher’s note: Undertaking any experimental protocol requires adherence to local institutional guidelines for laboratory safety and ethics.

## Graphical abstract

**Figure:**
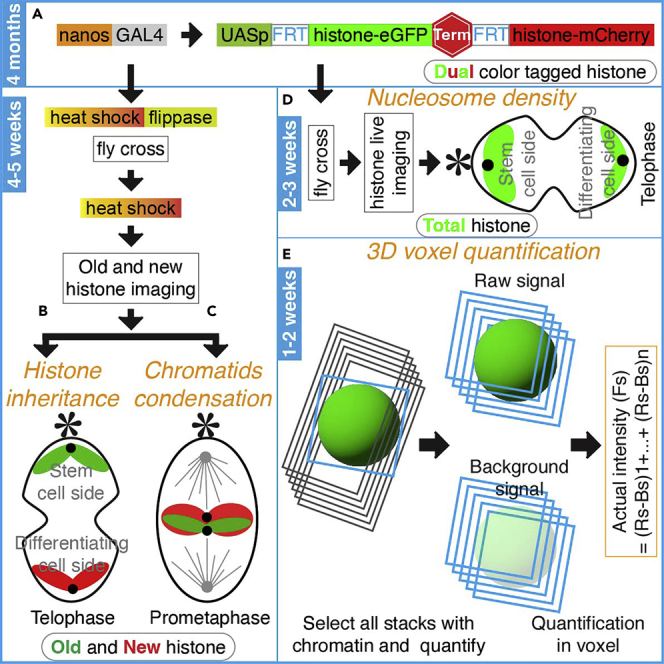

## Before you begin

The protocol described here provides details on studying ACD of stem cells, epigenetic inheritance and cell fates. Stem cells are unique cell types that can undergo asymmetric cell division (ACD) to produce two distinct daughter cells. Studies have shown that extrinsic cues and intrinsic factors can regulate ACD. Previously, our studies have revealed that the preexisting (old) and newly synthesized (new) histones segregate asymmetrically during ACD of *Drosophila* male germline stem cells (GSCs). Importantly, the old histone is predominantly inherited by the stem daughter cell and the new histone is enriched in the differentiating daughter cells. This asymmetric histone inheritance is critical for GSC maintenance and differentiation. Recently, we have identified several unique chromatin features of *Drosophila* male GSCs during ACD, which regulate distinct cell fates and differential cell cycle progression in the resulting daughter cells, including differential sister-chromatid condensation in mitosis, which results in asymmetric chromatin accessibility to replication initiation factors and asynchronous cell cycle progression in the daughter cells. In addition, we have discovered that sister chromatids in GSCs have asymmetric nucleosome density, with the old histone-enriched sister chromatids having higher nucleosome density compared to the new histone-enriched sister chromatids.

Here we detailed about developing genetic tools, *in vivo* live imaging and quantification methods to study ACD of stem cells. In summary, we propose that asymmetric nucleosome density could be used as a stem cell marker to distinguish stem cells at single-cell resolution, which could be widely appliable to many other systems including other stem cell lineages and asymmetrically dividing cells during development of multicellular organisms*.* These procedures will establish a new method for studying the ACD of stem cells and provide an imaging-based approach to study epigenome in the context of cell cycle.

Before quantifying histone inheritance patterns and chromatin status, develop transgenic *Drosophila* lines carrying fluorescence tagged histones and generate flies with appropriate genotypes for imaging experiments. In addition, acquire high spatial resolution or super-resolution fixed or live cell images for germline stem cells (GSCs) and spermatogonia cells (SGs), which serve as a control for GSCs.

### Preparation for transgenic Drosophila lines

**Timing: ∼ 3–4 months**

**Figure 1:**
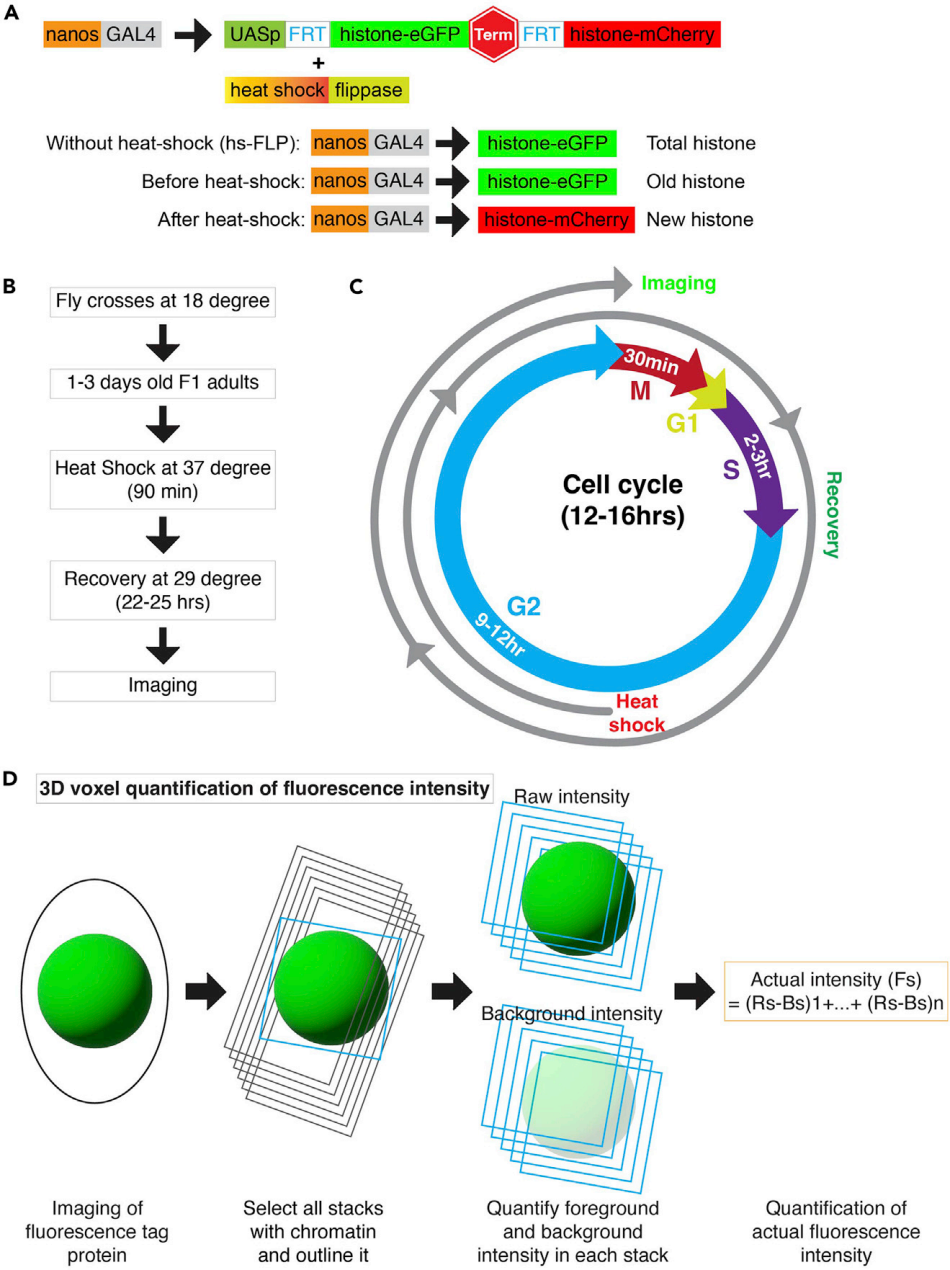
Genetic tool to distinguish old and new histone epigenome (A) Schematic of dual-color tag histones (first tag eGFP and the second tag mCherry) using *UASp-FRT-H3-EGFP-PolyA-FRT-H3-mCherry* construct. We have also developed a tag swapped version; first tag mCherry and the second tag eGFP (not shown here) using *UASp-FRT-H3-mCherry-PolyA-FRT-H3- EGFP* construct. Expression of flippase upon heat shock gives a temporal regulation of tag switching as indicated. In the heat shock scheme: after heat shock, the old histone would be eGFP tagged and the new histone would mCherry tagged. *nanos-GAL4* is an early germ cell driver. In the absence of heat shock flippase, only eGFP tagged histone would express. (B) Schematic of fly crosses for expression of dual-color histone. (C) Schematic of cell cycle map depicting heat shock timing and recovery duration for distinguishing old and new histone. (D) Diagrammatic representation of 3D voxel quantification of fluorescence intensity.

***Note:*** All core histones (H3, H4, H2A and H2B) or histone variants can be tagged with dual-color fluorescence for the study and one can choose the histone of interest to study using this method.

1. Develop dual-color fluorescence tagged histone transgenic fly lines using standard P-element techniques. Example: *UASp-FRT-Histone-EGFP-PolyA-FRT-Histone-mCherry* and *UASp-FRT-Histone-mCherry-PolyA-FRT-Histone-EGFP* (Figure 1A).

2. Genotype flies to check the copy number of the transgene; keep the lines with a single copy insertion for the following experiments. Map the chromosomal location of the transgene.
3. Develop or obtain a cell type-specific driver to drive the transgenic histone expression in your cell of interest, such as the *nanos-GAL4* driver (Tran et al., 2012; Van Doren et al., 1998) to drive transgene expression in GSCs and early-stage germ cells.
4. Develop or obtain the fly line carrying the *hs-FLP* transgene (heat shock promoter driving Flippase), which can switch expression of the fluorescence tagged histones when needed.

***Note:*** Upon heat shock (at 37°C for 90 min), Flippase in turned on and cleave the upstream tag by FRT recombination to express the downstream tag (Figure 1A).

5. Develop or obtain the fluorescence tagged α-Tubulin transgenic fly line.
6. Generate the following fly stocks: (i) Balanced or homozygous fly lines carrying the dual-color fluorescence tagged histone transgene, (ii) Balanced or homozygous fly line carrying the *hs-FLP; nanos-GAL4* transgenes, and (iii) Balanced or homozygous fly line carrying the *nanos-GAL4; UASp-tubulin-GFP* transgenes.

***Note:*** Balancers are used to trace transgenes during fly crosses. Notably, balancers themselves may cause some phenotype, therefore, screen for flies without balancers in your experimental flies.

### Crossing Drosophila lines

**Timing: ∼ 3–4 weeks**

7. Cross dual-color fluorescence tagged histone male flies, *UASp-FRT-Histone-mCherry-PolyA-FRT-Histone-EGFP*, with *hs-FLP; nanos-GAL4* female flies, to investigate histone inheritance pattern and sister-chromatid condensation dynamics. Grow these flies at 18°C (Figure 1B).
8. Cross *UASp-Histone-mCherry* flies with *nanos-GAL4; UAS-tubulin-GFP* flies, to investigate nucleosome density. Grow these flies at 25°C.

***Note:*** For single color histones expression, use dual color tagged histones (*UASp-FRT-Histone-mCherry-PolyA-FRT-Histone-EGFP*) without heat shock-induced flippase so as only mCherry tagged histone would be expressed. Alternatively, single-colored histone transgenic lines can be developed, such as the *UASp-H3-mCherry* line, which is available upon request.

9. Collect the 1–3-day old adult male flies for the following imaging experiments.

### Preparation of live imaging medium

**Timing: ∼ ½ day**

10. Prepare a standardized live cell imaging cocktail for respective tissue, cell or model system.

***Note:*** We have standardized for *Drosophila melanogaster* testes and can be used for this system (Ranjan and Chen, 2021).

## Key resources table

**Table:** 

REAGENT or RESOURCE	SOURCE	IDENTIFIER
**Experimental models: organisms/strains**		

*D. melanogaster*. *UAS-α-tubulin-GFP*	Bloomington *Drosophila* Stock Center	RRID: BDSC_7373
*D. melanogaster*. *hs-flp*	Bloomington *Drosophila* Stock Center	RRID: BDSC_26902
*D. melanogaster. nos-Gal4*	Kindly provided by Mark Van Doren, Johns Hopkins University, MD, USA	N/A
*D. melanogaster*. *nos-Gal4; tubulin-Gal80*^*ts*^	Kindly provided by Yukiko Yamashita, Whitehead Institute, MIT, USA	N/A
*D. melanogaster*. *UASp-FRT-H3-mCherry-FRT-H3-EGFP* or *UASp-FRT-H3-EGFP-FRT-H3-mCherry*	Our lab has developed	N/A
*D. melanogaster*. *UASp-FRT-H4-mCherry-FRT-H4-EGFP*	Our lab has developed	N/A
*D. melanogaster*. *UASp-FRT-H2A-mCherry-FRT-H2A-EGFP*	Our lab has developed	N/A

**Chemicals, Peptides, and Recombinant Proteins**		

FBS	Sigma	Cat#F2442
Penicillin/Streptomycin	Sigma	Cat#P0781
Mounting Medium	Vector	Cat# H-1400
BSA	Cell Signaling Technology	Cat#9998
Formaldehyde	Fisher Scientific	Cat#F79-500
Schneider Medium	Thermo Fisher Scientific	Cat# 21720024
Insulin	Thermo Fisher Scientific	Cat#12585014
FluoroDish	World Precision Instruments, Inc.	Cat#FD35PDL

**Software and algorithms**		

Adobe Illustrator CS6	Adobe	N/A
Prism 6	GraphPad	N/A
Fiji	NIH	N/A
EndNote	Clarivate Analytics	N/A
IMARIS	Bitplane	N/A

## Materials and equipment

### Stock solutions

#### Preparation of live cell imaging cocktail (live cell media)

Prepare Schneider’s *Drosophila* medium with 15% fetal bovine serum (FBS) and 0.6× penicillin/streptomycin. Adjust the pH to approximately 7.0. Add insulin to a final concentration of 200 μg/mL just before using the medium.

**Table:** 

Reagent	Final concentration	Amount
Schneider’s medium	N/A	8.5 mL
Fetal bovine serum	15%	1.5 mL
Penicillin/Streptomycin	0.6×	6 μL
Insulin	200 μg/mL	25 μL
Total	N/A	∼ 10 mL

***Note:*** This media is critical for maintaining normal cell division in the *Drosophila* testis during time-lapse imaging. Aliquot the media (without insulin) and store at 4°C for up to 5–6 months. Aliquot insulin separately and store at −20°C for up to a year. Mix aliquot of media and insulin just before sample preparation.

#### Microscope

• Live-cell imaging: Perform high spatial and temporal resolution live-cell imaging using Spinning Disk Confocal microscope.

***Note:*** All movies were taken using spinning disk confocal microscope (ZEISS) equipped with an evolve^-TM^ camera (Photometrics), using a 63× Zeiss objective (1.4 NA) at 29°C. The ZEN 2 software (ZEISS) was used for acquisition with 2 × 2 binning.

• Fix cell imaging: Perform high spatial resolution microscopy for fixed cell.

***Note:*** All fixed images were taken using Zeiss LSM 780 confocal microscope with 63× Zeiss objective (1.4 NA) oil immersion objectives. The ZEN 2 software (ZEISS) was used for acquisition.

## Step-by-step method details

### Part 1: Investigating histone inheritance pattern in live cells

**Timing: ∼ 3 months (for step 1)**

Dual-color fluorophore switchable genetic tool to study histone inheritance:

Dual color tagged histone transgenic flies, *UASp-FRT-Histone-EGFP-PolyA-FRT-Histone-mCherry*, and *hs-FLP; nanos-Gal4* flies were made. Dual color-tagged histone transgenic flies were used to distinguish between old and new histone. The *nanos-Gal4* driver was used to drive expression of histone in the early germline cells. The *hs-FLP* was used to flip the histone tag.

1. Use a dual color fluorescence tagged transgenic histone flies, for example, *UASp-FRT-Histone-EGFP-PolyA-FRT-Histone-mCherry* (Tran et al., 2012; Wooten et al., 2019; Ranjan et al., 2019) (Figure 1A).
2. Use a heat shock-induced genetic switch system, for example, the *hs-FLP* transgene allows flippase expression upon heat shock (Figure 1A).
3. Use a tissue and cell type-specific driver (cell/tissue of interest), for example, *nanos-Gal4* for expressing transgenes in GSCs and early-stage germ cells (Figure 1A).

**CRITICAL:***Histone-EGFP* followed by *PolyA* ensure Histone-EGFP expression in the absence of flippase. *Histone-EGFP-PolyA* flanked by two *FRT* sequences followed by *Histone-mCherry-PolyA* ensure cleavage of *Histone-EGFP-PolyA* and expression of Histone-mCherry in the presence of flippase. This facilitates a temporal control of tag switching upon heat shock (Figure 1A). Since this tag switching happens at the genetic level, it is an irreversible process.

#### Fly crosses

**Timing: ∼ 4–5 weeks (for step 4)**

Dual color tagged histone transgenic flies were crossed with the *hs-FLP; nanos-Gal4* flies to get progeny expressing both the *UASp-FRT-H3-EGFP-PolyA-FRT-H3-mCherry* and the *hs-FLP; nanos-Gal4*. The F1 progeny were used to heat shock to flip the histone tag (EGFP to mCherry) to distinguish old vs. new histones. Live cell imaging was performed to capture mitotic GSCs and SGs to determine old and new histones inheritance patterns in telophase.

4. Cross the *UASp-FRT-Histone-EGFP-PolyA-FRT-Histone-mCherry* male flies with the *hs-FLP; nanos-Gal4* female flies (Figure 1A).

***Note:*** It is important to use dual-color histone switchable system which can switch the expression of different tagged histones. Start separate crosses for different histone transgenes, e.g., H3, H4, and H2A.

5. Maintain fly crosses at 18°C incubator throughout development (Figure 1B).

***Note:*** Once the cross starts, do not take the flies outside the 18°C incubator, which could result in pre-flipping of the FRT-flanking histone tag.

6. Collect 1–3-day old adult F1 male progenies in a new vial (Figure 1B).
7. Submerge the vial underneath water up to the plug in a circulating 37°C water bath for 90 min (Figure 1B).

***Note:*** Make sure to put the vial properly in the water bath; the water level should be reaching at least the cotton plug to ensure a uniform temperature inside the vial. A water bath gives a more stable temperature and highly reproducible results compared to the air incubator.

**Figure 2:**
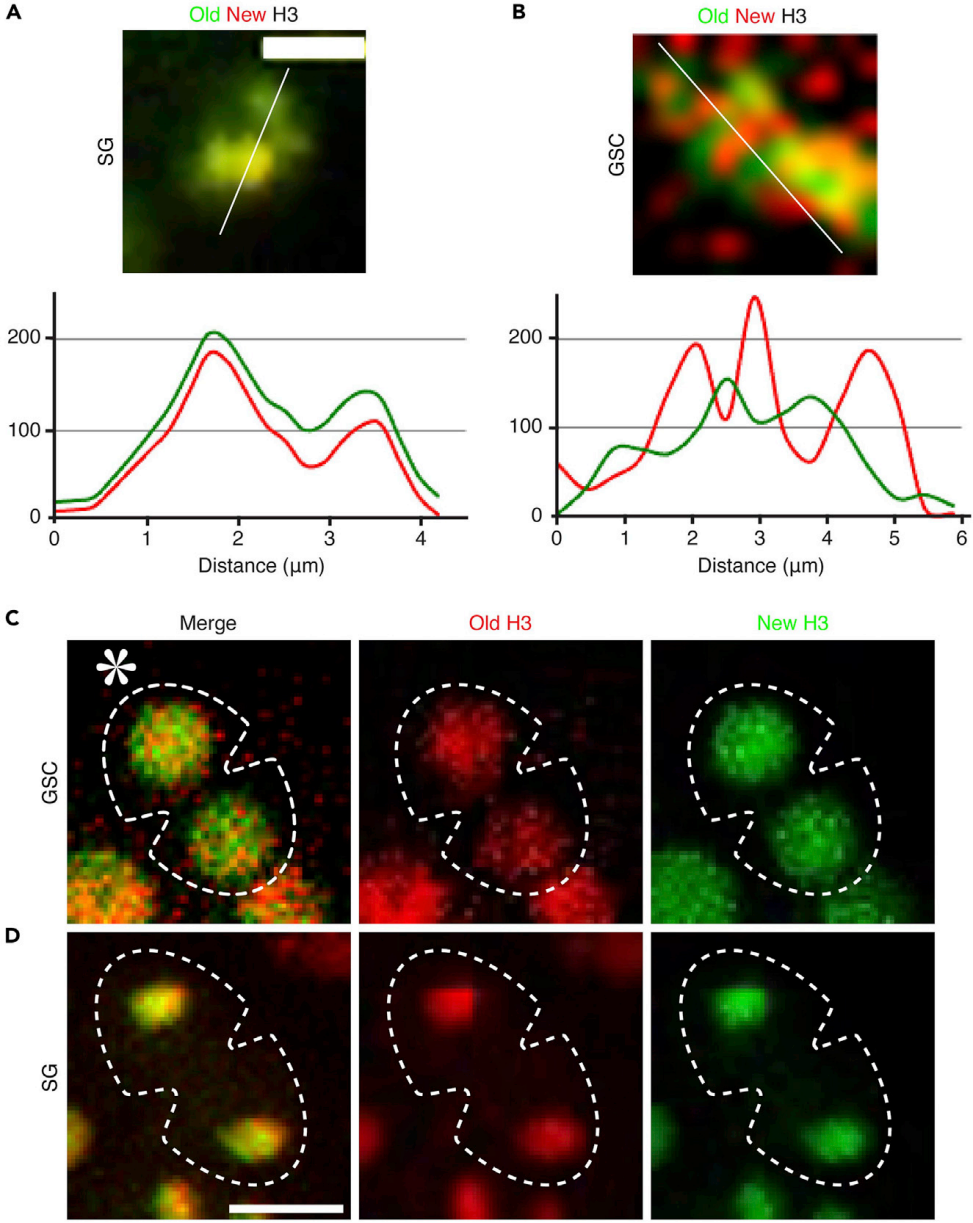
Tracking old and new histone dynamics and their inheritance in live tissue during ACD (A and B) A dual color histone tag, *UASp-FRT-H3-EGFP-PolyA-FRT-H3-mCherry,* was used to investigate histone dynamics in mitosis. (A) Representative image showing overlapping old and new histone H3 during SG’s mitosis. The line plot shows overlapping peaks for old and new histone regions in the genome in SG. (B) Representative image showing non-overlapping old and new histone H3 during GSC’s mitosis. The line plot shows mutually exclusive peaks in the genome in GSC. (C and D) A tag swapped version of dual color histone, *UASp-FRT-H3-mCherry -PolyA-FRT-H3-EGFP,* was used to investigate histone inheritance in telophase. (C) Representative image showing asymmetric old histone H3 (mCherry tag, red) segregation in telophase in GSC. (D) Representative image showing symmetric old histone H3 (mCherry tag, red) segregation in telophase in SG. Scale bar = 5 μm; Asterisks = niche (hub region).
12. Analyze histone incorporation pattern in mitosis using line plot.

***Note:*** Non-overlapping old and new histones would indicate biased incorporation of histone on sister-chromatids during S phase (Figures 2A and 2B).

8. Transfer heat-shocked flies into a new vial and recover them in a 29°C incubator for 22–25 h before dissection for imaging experiments (Figure 1C).
9. If performing live-cell imaging, dissect flies in a live imaging medium, clean and wash the testes with live imaging medium. Mount testes in the glass bottom dish, add 100–150μL of live imaging medium and cover the lid. Place the sample under the spinning disk confocal microscope [for details please check our previous method paper (Ranjan and Chen, 2021)].
10. Imaging: For live-cell imaging, use a high-resolution Spinning Disk Confocal microscope or high spatial resolution microscope [for detail check (Ranjan and Chen, 2021)].
11. Acquire high spatial and temporal resolution images (Figures 2A–2D).

13. Next, perform quantification (Figure 1D) of old and new histones in anaphase or telophase GSCs (Figures 2C and 2D).

**CRITICAL:** The recovery time depends upon cell cycle length for the cell types of your interest. It is critical to ensure that a complete S phase occurs during the recovery, when new histones are synthesized and incorporated, before visualizing old and new histone patterns in the subsequent mitosis (Figure 1C). Since *Drosophila* male GSCs has a prolonged G2 phase (9–12 h out of the 12–16-h cell cycle) (Lenhart and DiNardo, 2015) (Gadre et al., 2020), most GSCs are heat shocked during G2 phase. Therefore, 22–25 h are considered sufficient for the GSCs to go through a complete cell cycle (M-G1-S phases), before entering the subsequent M phase. Therefore, we standardize this as the right time point to investigate histone inheritance patterns in male GSCs, as described in the schematic diagram (Figure 1C).

#### Voxel quantification assay for histone inheritance

**Timing: ∼ one week (for step 14)**

To understand the old and new histone inheritance pattern during asymmetric stem cell division, perform 3D quantification of old and new histone inheritance in telophase.

14. Save raw images (as 2D Z-stacks) from live-cell imaging.
15. Open them on FIJI (ImageJ) software and draw the largest freehand circle that fits within the GSC or the SG to define histone-occupied chromatin region to include all fluorescent histone signals associated with the chromatin (Figure 1D).
16. Process the image to get the gray value of each pixel by using "display_pixel_values" plugins using FIJI (ImageJ) software.
17. Determine the sum of the gray values of all pixels in each Z-stack (‘‘RawIntDen’’).
18. Determine the sum of the gray values of background pixels in each Z-stack (‘‘RawIntDen’’).

***Note:*** Use the same freehand circle to determine the foreground fluorescence signal and the background signal to ensure the same area was used (Figure 1D). For background signal, use the hub region (niche) or any negative region in the tissue where fluorescence tagged protein is not expressed.

19. Calculate the actual gray values of the histone fluorescent signal pixels for each Z stack (Foreground Signal, Fs) by subtracting the gray values of the background signal pixels (Background Signal, Bs) from the gray values of the raw signal pixels (Raw Signal, Rs).

Fs = Rs - Bs.

20. Next, calculate the total amount of the histone fluorescent signals in the cell (Voxel) by adding the gray values of the fluorescence signal from all Z-stacks (Figure 1D).

The total amount of the fluorescence signal (Fs) in the nuclei with ‘n’ Z-stacks (Z1+…+Zn). Fs (Z1+…+Zn) = [(Rs-Bs)1+…+(Rs-Bs) n].

21. Use 15–25 pairs to determine the actual amount of histone in voxel in GSC-GB pairs and SG1-SG2 pairs in telophase.

***Note:*** The total amount of gray value would represent the total amount of histone protein in the cell (Voxel=quantification in the volume of the cell).

#### Analysis of histone inheritance

22. Calculate the old histone ratio, GSC to GB, and new histone ratio, GSC to GB. For control, calculate the old histone ratio, SG1 to SG2, and the new histone ratio, SG1 to SG2.
23. Plot the dot plot for all the datasets, determine the mean value and standard error of the mean (SEM), and calculate the statistical significance.
24. Determination of asymmetry: We define asymmetric inheritance of histone if mean value in GSCs is > mean + 1∗STD in control SGs. We define symmetric inheritance of histone if mean value in GSCs is < mean + 1∗STD in control SGs (Wooten et al., 2019; Ranjan et al., 2019).

***Note:*** Individual GSC-GB pairs and SG pairs can be assigned as symmetric or asymmetric using these criteria to determine the overall percentage of symmetric or asymmetric pairs to present in a histogram.

**CRITICAL:** To determine the histone inheritance pattern, old histone would be more reliable because no old histone synthesis and incorporation happens once the encoding sequences have been flipped out. To know the accurate difference in histone inheritance pattern, it is crucial to quantify the total amount of histone (without the tag switch) in a voxel (as described below in section 3).

### Part 2: Investigating sister chromatids condensation in mitosis (in live-cell and fixed cell)

#### Imaging old and new histone-enriched sister-chromatids

**Timing: ∼ 3–5 weeks (for step 25)**

Dual color tagged histone transgenic flies were crossed with the *hs-FLP; nanos-Gal4* flies to get progeny expressing both the *UASp-FRT-H3-EGFP-PolyA-FRT-H3-mCherry* and the *hs-FLP; nanos-Gal4*. The F1 progeny were used to heat shock to flip the histone tag (EGFP to mCherry) to distinguish old vs. new histones. Live cell imaging was performed to capture mitotic GSCs and SGs to distinguish chromatids enriched with old and new histones.

**Figure 3:**
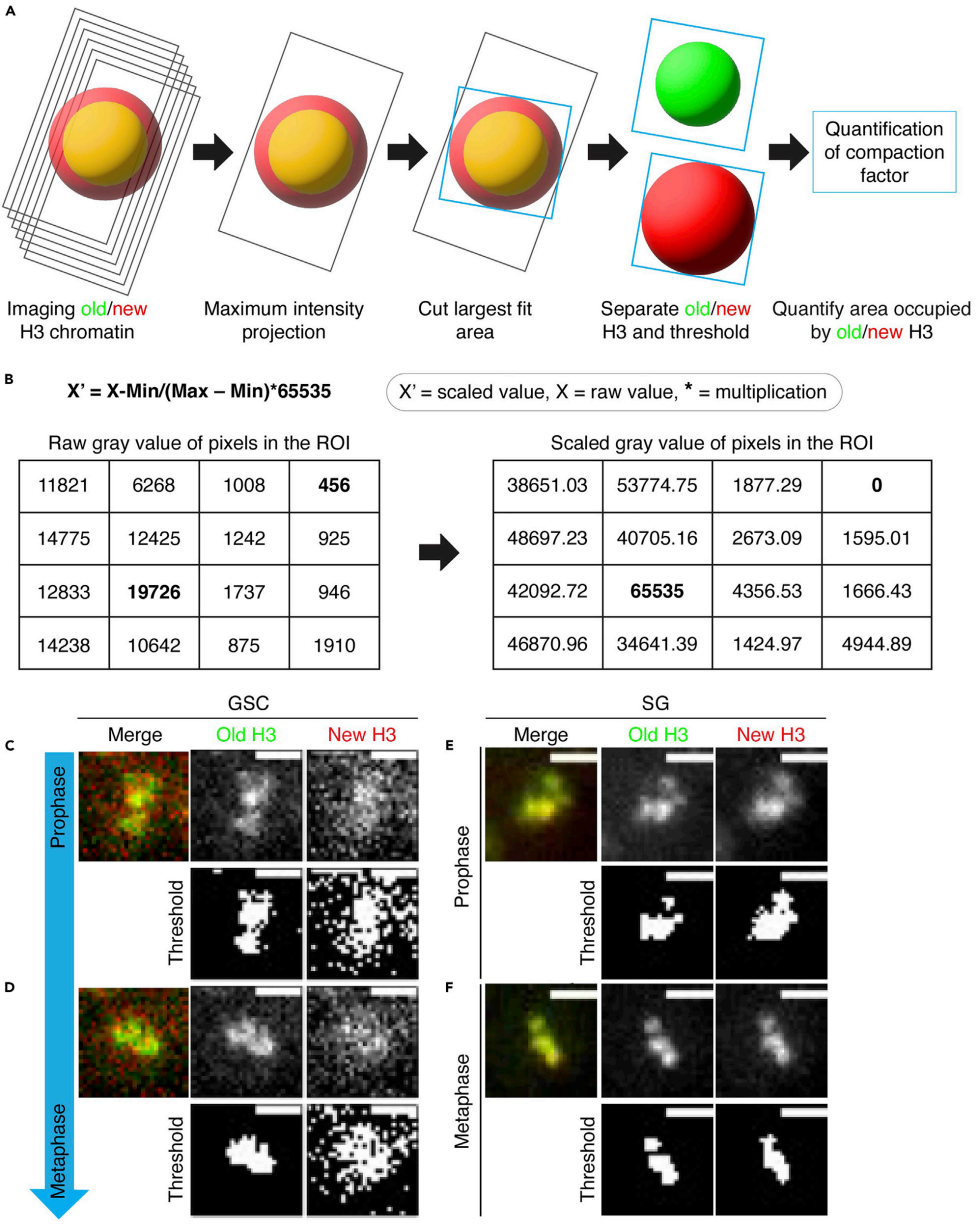
Quantification of sister-chromatids condensation in germline stem cells (A) Diagrammatic representation of quantification of sister-chromatids condensation in mitosis. Steps showing how to process images for old and new histone enriched chromatin condensation assay. (B) Showing a formula to re-scale pixels intensity between 0 to 65535 (for the 16-bit camera). Representative gray value of each pixel of the region of interest [(ROI), grid] before and after scaling. (C and D) Representative image of GSC showing asymmetric condensation of old and new histone H3 enriched chromatin during mitosis, prophase (C) to metaphase (D). The panel also shows individual channels before and after the threshold. (E and F) Representative image of SG showing symmetric condensation of old and new histone H3 enriched chromatin during mitosis, in prophase (E) and metaphase (F). The panel also shows individual channels before and after the threshold. Scale bar = 5 μm.

25. Use dual-color tagged histone H3 transgene line [as described above (Figure 1A)] to study sister chromatids condensation dynamics during mitosis.
26. Cross the *UASp-FRT-H3-EGFP-PolyA-FRT-H3-mCherry* male flies with *hs-FLP; nanos-GAL4* female flies, and maintain the cross at 18°C (Figure 1B).
27. Collect 1–3 day-old adult male flies (F1 progeny) and heat shock them in a 37°C water bath for 90 min. Recover them for 22–25 h at 29°C before dissecting them for experiments (Figure 1C).
28. Image GSCs and SGs for old histone H3-enriched chromatids and new histones H3-enriched chromatids using live-cell imaging (Ranjan and Chen, 2021) or fixed cell imaging (Wooten et al., 2019; Ranjan et al., 2019).
29. Collect all mitotic (late prophase to prometaphase) cells and perform the condensation assays (Figures 3B–3E).

#### Old and new histone-enriched chromatin condensation assay

**Timing: ∼ 1–2 weeks (for step 30)**

Condensation assay was performed to evaluate the condensation dynamics of old and new histone-enriched chromatin during mitosis.

30. Generate a maximum intensity projection for each GSCs and SGs (Figure 3A).
31. Draw a largest freehand circle that fits within the GSC or the SG to define histone-occupied chromatin area in the nucleus for analysis (Figure 3A).

***Note:*** (i) For live cell imaging, use histone signal to define chromatin region. (ii) For fix cells, use histone signal or Hoechst (DNA dye) to define chromatin region.

32. Determine the gray value of each pixel of the image by using "display_pixel_values" plugins using FIJI (ImageJ) software.
33. Individually scale the images of each GSC or SG nucleus, setting the minimum intensity to 0 and the maximum to 65,535 (for 16-bit range) (Figure 3A).
  Use the below formula to scale the image on excel sheet:

X′ = X-Min/(Max – Min)∗65535.

Where, X′ = scaled value, X = raw value, Min = minimum raw value in the region of interest (ROI), Max = maximum raw value in the ROI, ∗ = multiplication, 65535 = maximum gray value of a 16-bit camera.

34. Quantify the fluorescence intensity distribution by monitoring the flux of pixels at a threshold of 35% of the maximum intensity of the image (scaled intensity: 22,937) (Figures 3B–3E).

***Note:*** Before condensation, the signal is relatively homogeneous. As the chromosomes condense in mitosis, the histone fluorescence concentrates to a smaller area within the nucleus. Thus, the smaller area occupied by the chromatin could indicate compacted chromosomes.

35. Determine the condensation parameter for old histone H3-enriched regions and new histone H3-enriched regions by calculating the number of pixels above 35% threshold intensity (the condensation parameter).

***Note:*** The number of pixels represents the area occupied by the pixels above 35% threshold intensity in the nucleus (region of interest), and hence represents the area occupied by the chromatin.

36. Measure the relative condensation of old histone H3-enriched regions by calculating the ratio of condensation parameters of new histone H3-enriched regions to old histone H3-enriched regions in prometaphase for GSCs and SGs (Figures 3A–3E).

Compaction factor = number of pixels of new histone H3-enriched chromatin / number of pixels of old histone H3-enriched chromatin.

**CRITICAL:** Individual scaling of the image ensures that the fluorescence intensity distribution is independent of fluctuations due to photobleaching, changes in illumination intensity, or variations among samples.

#### Analysis of old and new histone-enriched chromatin condensation

37. Use 15–25 GSCs and SGs, and determine the relative condensation of old histone H3-enriched regions compared to the new histone H3-enriched regions.
38. Generate a dot plot for all datasets and determine the mean value and standard error of the mean (SEM), and calculate statistical significance.
39. Defining condensation statuses between sister chromatids: Differential sister chromatids condensation: if mean value of compaction factor in GSCs is > mean + 1∗STD in control SGs. Equal sister chromatids condensation: if mean value of compaction factor in GSCs is < mean + 1∗STD in control SGs (Ranjan et al., 2022).

***Note:*** Individual GSCs or SGs can be assigned differential or equal condensation to determine the percentage of asymmetric or symmetric cells (Ranjan et al., 2022).

**CRITICAL:** Averaging 15–25 GSCs or SGs for quantification of compaction factors is essential to normalize signal intensity variations that could arise from differences among cell and tissue types and the microscope settings. Importantly, averaging minimizes the contribution of spatial fluctuations and emphasizes the global changes in sister chromatid structure.

### Part 3: Quantification of nucleosome density on sister chromatids

#### Live imaging of histones for quantification of nucleosome density

**Timing: ∼ 15–20 days (for step 40)**

Dual color tagged histone transgenic flies were crossed with the *nanos-Gal4, tubulin-GFP* flies to get progeny expressing single color histone (H3-mCherry) and *α-tubulin-GFP*. The F1 progeny do not need to heat shock as we need to quantify the total amount of histone. Live cell imaging was performed to capture mitotic GSCs and SGs.

40. Cross mCherry tagged histones (H3, H4 or H2A) fly line: *UASp-Histone-mCherry* with *nanos-GAL4, α-tubulin-GFP* fly line and grow them at 25°C.

***Note:*** α-tubulin-GFP was used to track mitotic cells in live imaging. One can use dual color tagged histone, UASp-FRT-H3-mCherry-PolyA-FRT-H3-EGFP, flies without flipping histone tag using heat shock treatment.

**Figure 4:**
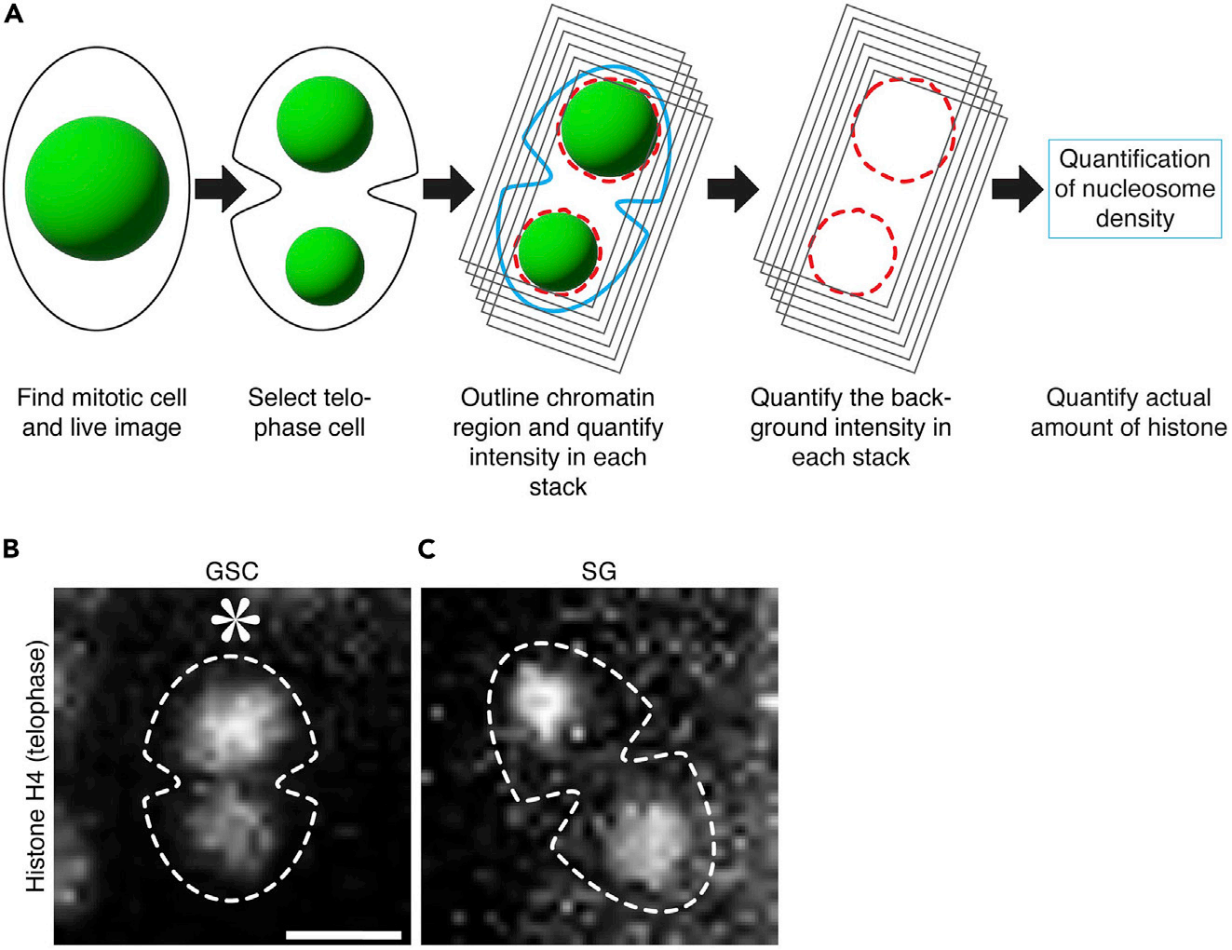
Analysis of nucleosome density on sister-chromatids in germline stem cells (A) Diagrammatic representation of quantification of nucleosome density of sister-chromatids in telophase. Steps showing how to process images for 3D nucleosome density quantification. Also shows a formula to calculate the relative nucleosome density of the sister chromatids. (B) Representative image of GSC-GB pair showing asymmetric nucleosome density on sister-chromatids in telophase. (C) Representative image of SG1-SG2 pair showing symmetric nucleosome density on sister-chromatids in telophase. Scale bar = 5 μm; Asterisks = Niche (hub).

***Note:*** High spatial and temporal resolution images would give the most accurate nucleosome density profile.

41. Collect 1–3 day old adult male flies for live cell imaging.
42. Acquire high spatial and temporal resolution time-lapse movies of GSCs and SGs and use telophase cells for quantifications (Figures 4B and 4C) (Ranjan et al., 2022).

#### Quantification of total histone fluorescence signal (voxel quantification)

**Timing: ∼ 1–2 weeks (for step 43)**

Quantify the total amount of histone in telophase to determine nucleosome density on sister chromatids segregated into the stem daughter cell and differentiating daughter cell.

43. Save raw images (as 2D Z-stacks) as TIF images from live cell imaging.
44. Open the image on FIJI (ImageJ) software and draw the largest freehand circle that fits within GSCs or SGs to define histone occupied chromatin region to ensure all fluorescence signals associated with the chromatin were measured (Figure 4A).

***Note:*** Use histone fluorescence signal to mark chromatin region.

45. Process the image to get the gray value of each pixel by using "display_pixel_values" plugins using FIJI (ImageJ) software.
46. Determined the sum of the gray values of the fluorescence signal of each pixel in each Z-stack in the image (‘‘RawIntDen’’).
47. Determined the sum of the gray values of background pixels in each Z-stack in the image (‘‘RawIntDen’’).

***Note:*** Use the same freehand circle to determine the foreground fluorescence signal and the background signal to ensure the same area was used. For background signal, use the hub region (niche) or any negative region in the tissue where fluorescence tag protein is not expressed.

48. Calculate the actual gray values of the fluorescence signal pixels for each Z stack (Foreground Signal, Fs) by subtracting the gray values of the background signal pixels (Background Signal, Bs) from the gray values of the raw signal pixels (Raw Signal, Rs).

Fs = Rs - Bs.

49. Next, calculate the total amount of the fluorescence signal in the nuclei by adding the gray values of the fluorescence signal from all Z-stacks (Figure 4A).
  The total amount of the fluorescence signal (Fs) in the nuclei with ‘n’ Z-stacks (Z1+…+Zn).
  Fs (Z1+…+Zn) = [(Rs-Bs)1+…+(Rs-Bs) n].
  ***Note:*** The total amount of gray value would represent actual amount of histone protein in the nucleus [quantification in volume (Voxel)].

50. Use the below formulas for the calculation:

Number of nucleosomes in the genome = ½ amount of histone.

Average nucleosome density = Number of nucleosome / the genome length.

***Note:*** Two copies of each core histones (2× H3, 2× H4, 2× H2A, and 2× H2B) are present in the nucleosome. Thus, the amount of nucleosomes would be half the amount of histones.

51. Determine the ratio of nucleosome density between sister chromatids segregated toward the stem daughter cells (GSC side) and differentiating daughter cells (GB side) in telophase (Figure 4A).

Nucleosome density ratio (GSC/GB) telophase = (Nucleosome number / length of DNA)_GSC side_ / (Nucleosome number / length of DNA)_GB side_

= (Nucleosome number)_GSC side_ / (Nucleosome number)_GB side_

= (½ total amount of histones)_GSC side_ / length of DNA **/** (½ total amount of histones)_GB side_ / length of DNA

= (total amount of histones)_GSC side_**/** (total amount of histones)_GB side_.

***Note:*** Ratio of total amount of histone in telophase would reflect relative nucleosome density between segregated sister chromatids (Ranjan et al., 2022).

52. For control, determine the ratio of nucleosome density between the sister chromatids segregated in telophase SGs.

Nucleosome density (SG1/SG2) telophase = (total amount of histones)_SG1 side_ **/** (total amount of histones)_SG2 side_.

***Note:*** Orient SG toward the fusome structure (SG1=towards fusome and SG2 = away from fusome).

**CRITICAL:** The length of DNA for both sets of sister chromatids should be equal; therefore, the amount of histone would be directly correlated to genome-wide average nucleosome density.

#### Analysis of nucleosome density

53. Use 10–15 GSC-GB pairs and 10–15 SG1-SG2 pairs to calculate the nucleosome density.
54. Generate the dot plot for all the datasets and determine the mean value and standard error of the mean (SEM) and calculate statistical significance.
55. Defining nucleosome density status of the sister chromatids in telophase: we define asymmetric nucleosome density: if the mean value in GSC-GB pairs is > mean + 1∗STD in control (SG1-SG2 pairs). We define symmetric nucleosome density: if the mean value in GSC-GB pairs is < mean + 1∗STD in control (SG1-SG2 pairs) (Ranjan et al., 2022).

**CRITICAL:** In telophase, sister chromatids segregate to the opposite pole and are easy to quantify. Thus, the quantification of nucleosome density at this stage would reflect the average nucleosome density on each set of the sister chromatids.

## Expected outcomes

Using this protocol, we have established an optimized method to track histone inheritance patterns, sister-chromatid condensation dynamics, and sister chromatids nucleosome density during ACD in live cells within intact tissues. This method comprises three major parts:

The first part of this protocol is adapted from previous assays of asymmetric histone inheritance patterns in *Drosophila* male GSCs (Ranjan et al., 2022; Tran et al., 2012; Wooten et al., 2019). We have established genetic tools to study inheritance patterns of different histones during ACD of male GSCs. In this protocol, we use dual-color fluorescence tagged histones (e.g., H3, H4, and H2A) to track old *versus* new histone distribution and inheritance patterns in mitosis. Histones H3 and H4 show a global asymmetric inheritance in *Drosophila* male GSCs [Figures 2C and 2D (Tran et al., 2012; Wooten et al., 2019)] and intestinal stem cells (ISCs) (Chen and Zion, 2020), whereas there is a local asymmetry in *Drosophila* female GSCs (Kahney et al., 2020) and mouse embryonic stem cells (Ma et al., 2020). However, H2A shows an overall symmetric inheritance pattern in *Drosophila* male and female GSCs as well as ISCs. Conversely, symmetrically dividing SG cells do not show such an asymmetric inheritance of H3 or H4. Together, our quantification revealed a molecular and cellular specificity of histone inheritance (Tran et al., 2012; Wooten et al., 2019).

The second part of this protocol is adapted from previous assays of asymmetric sister-chromatid condensation in *Drosophila* male GSCs (Ranjan et al., 2022; Maddox et al., 2006). In this protocol, we used the dual-color tagged histone transgene system to track the condensation dynamics of the old versus new histone-enriched sister chromatids during mitosis using live cell [Figures 3B–3E; (Ranjan et al., 2022)] and fixed cell imaging. We found that sister chromatids in GSCs show differential condensation during mitosis (Figures 3B and 3C). Conversely, SGs show symmetric sister-chromatid condensation (Figures 3D and 3E). Together, our quantification revealed a cellular specificity of the differential condensation of sister chromatids during ACD (Ranjan et al., 2022).

The third part of this protocol is adapted from previous assays of asymmetric nucleosome density in *Drosophila* male GSCs (Ranjan et al., 2022). In this protocol, we use mCherry tagged histone H3, H4, and H2A to track nucleosome density on sister chromatids in telophase (Figures 4B and 4C; (Ranjan et al., 2022). Our results showed that the sister chromatids inherited toward the GSC have 30%–40% more nucleosome than the sister chromatids inherited by the GB. Such asymmetric nucleosome density was not observed in SGs. Together, our quantification methods have shown a cellular specificity of the asymmetric nucleosome density phenomenon (Ranjan et al., 2022).

After performing a series of quantification and characterization of chromatin features in male GSCs (Ranjan et al., 2022), we propose that the nucleosome density asymmetry between the two sets of sister chromatids can be used as a marker to distinguish stem cells. Importantly, visualization and quantification of such phenomena require no unique reagent or techniques; therefore, it could be widely used in different systems across species.

***Note:*** Reagents for live cell imaging could vary among different organisms and model systems, therefore it needs to be optimized. Also, dual color histone tagging system could be different for different systems and need to be optimized accordingly, for example, photoconvertible Dendra2 was used in the induced asymmetrically dividing mouse embryonic stem cells (Ma et al., 2020).

Altogether, this protocol is suitable for other types of stem cells to explore histone inheritance patterns, chromatin condensation dynamics, and nucleosome density differences in cells from either cultured conditions or in the intact tissue context *in vivo*.

## Quantification and statistical analysis

1. Measurement of old *versus* new histone inheritance.
  We developed a voxel quantification method using a dual-color tagged histone system to distinguish old *versus* new histones and quantify their inheritance patterns (Figure 1D).
  a. For each focal plane of the cell, the gray value of an individual voxel for a channel (old or new histone channel) is normalized by the gray value of the background of that channel (Figure 1D).
    Thus, the total amount of fluorescence signal (Fs) in nuclei with ‘n’ Z-stacks (Z1+…+Zn); Fs (Z1+…+Zn) = [(Rs-Bs)1+…+(Rs-Bs) n].
  b. The ratio for each channel (old or new histone) is calculated in telophase on the normalized gray values of the voxel.
    GSC/GB_telophase_ = [(Rs-Bs)1+…+(Rs-Bs) n]_GSC_ / [(Rs-Bs)1+…+(Rs-Bs) n]_GB_.
  c. The overall mean value has been calculated, and the inheritance pattern has been determined for both old and new histones. Histone inheritance is asymmetric if the mean value is > mean + 1∗STD in control SGs. Whereas histone inheritance is symmetric if the mean value is < mean + 1∗STD in control SGs.
    **CRITICAL:** The voxel quantification method was developed given three premises. (1) Transgene with fluorescence tagged histone is single copy and flipping is induced by heat shock, (2) the incorporation of new histones is genome-wide (the cell has gone through a complete S-phase after heat shock-induced genetic switch), and (3) Since different channels have variabilities in many aspects and cannot be compared directly, voxels of different channels were normalized respectively in each focal plane for each channel (Figure 1D).

2. Measurement of the condensation dynamics of sister chromatids.
  We developed an area-based quantification method using a dual-color tagged histone system to quantify the condensation dynamics of the old *versus* new histone-enriched sister chromatids.
  a. For each cell, generate a maximum intensity projection and draw the largest circle that fits within the cell to outline the area occupied by histones. Determine the gray value of each pixel in the image and individually scale the images of each cell by setting the minimum intensity to 0 and the maximum to 65,535 (for 16-bit range) to normalize variations (Figure 3A).
  b. Determine the percentage of pixels above the 35% threshold intensity (it reflects the area occupied by pixels with > 35% normalized intensity) for old and new histone-enriched regions. The compaction factor is the ratio between the area occupied by the new histone-enriched and the old histone-enriched regions.
  c. The overall mean value of the compaction factor was calculated, and the condensation states of the old and new histone-enriched regions was determined. It is a differential condensation if the mean value of the compaction factor is > mean + 1∗STD in of the control SGs. It is an equal condensation if the mean value of the compaction factor is < mean + 1∗STD in the control SGs (Ranjan et al., 2022).
    **CRITICAL:** The area-based quantification method was developed given three premises: (1) As chromosomes condense during mitosis, they occupy less area; therefore, determining the area occupied by the histones would reflect the compaction state of the chromosomes. (2) As different channels exhibit variability in many aspects and cannot be directly compared, the relative compaction status between the old and new histone-enriched regions would normalize the variabilities among different time points in live imaging. (3) In male GSCs, old histones are enriched on one set of sister chromatids, and the new histones are enriched on the other set. Therefore, quantifying old and new histone-enriched regions would give a relative condensation state difference between the two sets of sister chromatids.

3. Measurement of the nucleosome density on sister chromatids in GSCs.
  We developed a nucleosome density assay using live cell imaging technique to measure nucleosome density difference between sister chromatids.
  a. Acquire high spatial and temporal resolution images and save un-scaled TIF images, and draw a large freehand circle to include the entire region occupied by the histone signals. Determine the sum of the gray values of the fluorescence signal of each pixel in each Z-stack and determine the sum of gray values of background pixels in each Z-stack of the image (Figure 4A).
  b. Subtract the gray values of the background signal pixels from the gray values of the raw signal pixels to get the real fluorescence signal of histones. Next, calculate the total amount of the fluorescence signal in the voxel by adding the gray values of the real fluorescence signal from all Z-stacks.
    The Real fluorescence signal in the voxel = [(Rs-Bs)1+…+(Rs-Bs) n]
  c. Determine the ratio of nucleosome density between the set of sister chromatids segregated toward the stem daughter side (GSC side) and the set by the differentiating daughter side (GB side) in telophase. The ratio of the total amount of histone segregated in GSCs and GBs at telophase would give the relative nucleosome density difference between sister chromatids in these two staged germ cells.
    Nucleosome density ratio (GSC/GB)_telophase_ = (total amount of histones)_GSC side_**/** (total amount of histones)_GB side_
  d. Determine the mean value of all datasets and define the nucleosome density status between sister chromatids. If the mean value in GSC-GB pairs is > mean + 1∗STD in control SG1-SG2 pairs, it will represent asymmetric nucleosome density. If the mean value in GSC-GB pairs is < mean + 1∗STD in control (SG1-SG2 pairs), it would represent symmetric nucleosome density (Ranjan et al., 2022).
    **CRITICAL:** The method was developed given three premises: (1) The imaged and quantified histones are part of the nucleosomes on sister chromatids. (2) The ratios of the total amount of histone on sister chromatids would reflect the relative nucleosome density, given that other factors in converting the amount of histones to genome-wide average density of nucleosomes are constant. (3) Variations among individual replication-dependent histones (e.g., H3 *versus* H4) are due to the presence of histone variants (e.g., H3.3) and its replication-independent mode of incorporation.

## Limitations

For the current protocol, several improvements could be made to further explore the relationship between histone inheritance, chromatin condensation, and nucleosome density to characterize stem cell features and to understand the process of cell fate determination.

We found asymmetric inheritance of old H3 and H4 in *Drosophila* male GSCs (Wooten et al., 2019; Tran et al., 2012; Wooten et al., 2020; Ranjan and Chen, 2022). In addition, we also reported global histone H3-H4 asymmetry in *Drosophila* ISCs (Chen and Zion, 2020) and local histone H3-H4 asymmetry in *Drosophila* female GSCs (Kahney et al., 2020) and induced mouse embryonic stem cells (mESCs) (Ma et al., 2020). These observations indicated that different systems regulate epigenome inheritance during ACD differently; it can be global or local at specific gene loci. While our quantification methods are sensitive to global histone inheritance, it does not give out gene-specific information. Thus, to decipher the histone inheritance at specific genomic loci, more technologies, such as TIGER-FISH (Kahney et al., 2021), CAS- FISH (Deng et al., 2015), CARGO-FISH (Gu et al., 2018), or dCas9-based methods (Tycko et al., 2016; Slesarev et al., 2019; Tadić et al., 2019), need to be used or developed to target candidate gene loci along with old and new histone occupancy information.

Currently our live cell imaging scheme for dual color histone tag lack fluorescent markers for determining cell cycle stages, which makes it difficult to precisely track germ cells at different cell cycle stages in live cells. Therefore, addition of a BFP-α-Tubulin transgene with dual color histone tags will be ideal for tracking chromatin landscape with precise cell cycle interpretation. However, addition of a third fluorescent marker could make the live cell imaging challenging, but it is feasible and could provide a complete picture of chromatin landscape throughout cell cycle.

We reported differential sister chromatid condensation during mitosis in *Drosophila* male GSCs. However, we do not know whether this difference relates to the whole genome or at some specific domains. Thus, to decipher the global *versus* local condensation asymmetry, other technologies such as super-resolution live snapshot (SRLS) (Ranjan and Chen, 2021) need to be applied to understand the chromosomal condensation patterns.

We reported asymmetric nucleosome density between sister chromatids during ACD of *Drosophila* male GSCs. However, we do not know whether this difference applies to the entire genome or at particular genomic loci. Thus, other technologies, such as ATAC-see (Chen et al., 2016), ATAC-PALM (Xie et al., 2020), ATAC-seq (Sato et al., 2019), and CUT&Tag (Kaya-Okur et al., 2019), need to be applied or developed to understand the pattern of nucleosome density asymmetry.

## Troubleshooting

### Problem 1

Variation in histone inheritance pattern (part 1).

### Potential solution

Ensure that only one copy of the fluorescently tagged histone transgene is inserted into the genome; more than one copy could give variability in inheritance patterns. Also, ensure that the cells go through at least one S-phase after flipping the fluorescence tag to ensure that new histones are effectively incorporated into the duplicated genome. If the cell did not go through the S-phase and the new histone was not incorporated, it would be difficult to distinguish between old and new histone. Therefore, it is essential to optimize the recovery time after heat shock-induced genetic switch, which could generate variations in inheritance patterns. The recovery time depends on the length of the cell cycle and must be optimized in different systems. For *Drosophila* male GSCs (cell cycle: 12–16 h), we found that the second mitosis after heat shock with ∼22–25-h recovery time is optimal (Figure 1C). If the cell cycle length is very short, a photoconvertible fluorescence tag can be used, such as Dendra2. The Dendra2 protein can be photoconverted (green to red) using 405 lasers within a minute, which helps distinguish old histone (red) from new histone (green) (Ma et al., 2020).

### Problem 2

Problem in visualizing sister chromatid condensation (part 2).

### Potential solution

Our quantification methods using prophase and prometaphase cells quantify the condensation of the old *versus* new histone enriched chromosomal region without distinguishing them between the sister chromatids. The old *versus* new histone segregation patterns need to be studied using anaphase and telophase cells when sister chromatids are separated, for which refer to section 1 and 3.

In the future, an imaging based method, such as SRLS (Ranjan and Chen, 2021) combined with mitotic chromosome spreading technique, could be developed to determine the precise differences between sister chromatids even in prophase, prometaphase and metaphase cells.

### Problem 3

Variation in nucleosome density with different histones (part 3).

### Potential solution

We used core histones (e.g., H3, H4 and H2A) to determine the asymmetry in nucleosome density on the sister chromatids and reported a 30%–40% asymmetry in nucleosome density. However, we also observe variations of about ∼10% among histones, such as between H3 and H4. We speculate that this is due to the presence of histone variants (such as H3.3 and CENP-A). Therefore, we propose to use histone H4, which has no known variants, for the determination of nucleosome density. In addition, we have reported that the differentiating daughter side enters the next S-phase early, which might start incorporating new histone prior to the stem cell side. Therefore, after telophase, the quantifications of nucleosomes may change due to asynchronous incorporation of new histones, thus we propose to perform quantification in telophase cells.

## Resource availability

### Lead contact

Further information and requests of reagents can be directed to and fulfilled by the lead contact, Xin Chen (xchen32@jhu.edu).

### Materials availability

All Histone transgenic *Drosophila* lines used in this study are available upon request from the lead contact, Rajesh Ranjan (rranjan4@jhu.edu); and Xin Chen (xchen32@jhu.edu).

## Data Availability

The manuscript contains all quantification codes used for histone inheritance, chromatin condensation measurement, and nucleosome density measurement.
